# Transportation infrastructure and economic growth: Evidence from "new nighttime light data" in the Yangtze River Delta

**DOI:** 10.1371/journal.pone.0306477

**Published:** 2024-08-02

**Authors:** Chunfang Huang, Hai Zhu, Meng Su

**Affiliations:** 1 School of Business, Jiangsu Second Normal University, Nanjing, China; 2 School of Business, Nanjing University, Nanjing, China; The University of Tokyo, JAPAN

## Abstract

The enhancement and improvement of China’s high-speed rail network plays a crucial role in promoting sustainable economic growth in the region. By utilizing "new nighttime light data" in China’s Yangtze River Delta from 2003 to 2018, this paper investigates the impact of HSR on economic growth using a multiperiod difference-in-differences (DID) model. The operations of high-speed rail have a significant and positive impact on economic growth, which often becomes more apparent with a certain time delay. The operations of high-speed rail have a significant positive impact on the development of large, mega, and super-cities, with this impact becoming more pronounced as the size of the city increases. Furthermore, high-speed rail operations have a significant influence on the economic growth of cities that largely rely on secondary and tertiary industries, in contrast to the primary industry. The promotion of economic growth by high-speed rail is primarily achieved through three transmission channels: low carbon emissions, labor force agglomeration, and innovation. Over time, high-speed rail will progressively reduce economic disparities between regions and facilitate a trend towards regional economic coordination or convergence. This study makes a valuable contribution to the exploration of pathways towards achieving economic growth.

## 1. Introduction

As China’s development stages progress, there is a transition occurring in China’s economy from a high-speed growth model to a high-quality growth model. However, issues of uneven development and unsustainability are becoming increasingly prominent. Obstacles to the free flow of human capital across regions, the need to strengthen endogenous economic growth drivers such as technological innovation, persistent administrative barriers, and environmental pollution are all significant hindrances to achieving economic growth. Therefore, it is crucial for China to align national strategies with regional planning during the 14th Five-Year Plan period (2021–2025) to accomplish high-quality economic development and foster coordinated regional growth. China’s convenient and efficient transportation infrastructure plays a crucial role in connecting different circulation links between production and consumption, thereby strengthening interactive connections among regions. Moreover, the green transformation of China’s high-speed rail network has achieved significant strides in reducing carbon emissions, making a substantial contribution to unlocking the inherent potential for economic growth and fostering coordinated regional development. The study specifically focuses on the Yangtze River Delta region in China as the key study area, aiming to examine the relationship between high-speed rail operations and regional economic growth. This research makes a valuable contribution to examining the role of high-speed rail in enhancing economic growth and development. It helps alleviate barriers to sustainable economic growth and generates new impetus for high-quality economic development.

During the 1960s, there was a surge of interest among scholars in studying the correlation between the transportation infrastructure of developed countries and their economic growth. Fishlow [1] was one of the pioneering economists who conducted a systematic analysis of the causal relationship between railroad construction and economic expansion. Drawing on his research on 19th-century America, he concluded that railroad construction appeared to be a response to demand rather than a direct cause of regional population growth. Aschauer [2] conducted pioneering research on the relationship between transportation infrastructure and economic growth, arriving at a groundbreaking conclusion that transportation infrastructure had significant positive effects on economic growth. One of his key findings was that infrastructure output elasticity was determined to be 0.39. However, these empirical findings were subject to challenge by Holtz-Eakin [3], who claimed that the impact of highways on US economic growth was limited. Later, Lachler and Aschauer [4] initiated a new discourse, emphasizing that investment alone does not guarantee economic growth. With the advent of new economic geography, scholars such as Holl [5], Fujita et al. [6], and Oosterhaven & Knaap [7] examined the influence of transportation infrastructure on firm distribution and industrial layout from the perspective of spatial agglomeration economics. Their findings indicate that the enhancement of transport infrastructure can potentially disrupt the existing equilibrium between regional centrifugal and centripetal forces. However, Ghali [8] and Presbitero [9] argue that the direction of the impact of transport infrastructure on economic growth is uncertain, as it could have both positive and negative effects.

Improving inter-regional transport infrastructure not only fosters economic growth but also facilitates the spatial mobility of economic factors, thereby potentially altering the spatial distribution pattern of economic activities. One perspective argues that high-speed rail promotes the agglomeration of production factors from peripheral areas to central regions, thereby suppressing the economic development of peripheral regions. Preston & Wall [10] and Hall [11] argue that the spatial impact of high-speed rail lines is complex. The construction of high-speed rail may benefit large central cities that are connected to it, while the status of more peripheral cities may be threatened, even leading to a flow of factor resources from the cities along the way to central cities. Murakami & Cervero [12] examined commercial agglomerations near 17 stations of the Tokaido Shinkansen, 30 stations of the Northeast Corridor, and 25 stations of the California High-Speed Rail. They argue that high-speed rail is likely to bring greater economic benefits to knowledge-intensive enterprises, although these enterprises are mostly concentrated in large cities that are globally connected, potentially sacrificing the benefits of small and medium-sized urban areas. Faber [13] conducted a study on the national expressway network of China and found that the GDP growth of surrounding counties traversed by expressways would decline, while the impact on counties farther away from the expressways would weaken. This suggests that economic activities tend to shift from rural areas to closely connected urban areas, resulting in agglomeration in metropolitan areas. Another perspective argues that HSR accelerates the frequency of communication between peripheral cities and central cities. Peripheral cities receive the spillover of population and resources from central cities, which greatly benefits regional coordinated development. Baum-Snow et al. [14] demonstrate that both radial and ring roads facilitate the large-scale dispersal of population from central cities to the surrounding areas. Additionally, railways and ring infrastructure promote the dispersion of industrial production and labor. Jedwab & Moradi [15] conducted a study on the impact of railway development on output and population agglomeration in Ghana and Africa, and found that both output and population increased in grid units traversed by railways. Additionally, they found that railways had a positive impact on the output of adjacent grid units, with this impact decreasing as distance from the railway increased.

While many scholars have made valuable contributions from different perspectives, a consensus has not been reached on the economic impact of high-speed rail. This lack of consensus can be attributed to variations in research methodologies, estimation models, temporal scopes, and data origins. Therefore, in order to gain a deeper understanding of the welfare effects of high-speed rail on economic growth, more refined data and models are required. This article is based on the "New Night-time Light Data Set," which is the first global 500-meter resolution night-time light dataset from 2003 to 2018, proposed by Chen et al. [16], and referred to as "NPP-VIIRS-like NTL Data." This dataset offers a more objective, comprehensive, and accurate measurement of urban economic activity. The novelty of this article lies in its utilization of the more granular "New Night-time Light Data Set" to investigate the influence of high-speed rail on economic growth. By doing so, it enhances our understanding of the welfare implications of high-speed rail and addresses a research gap in the field.

The research findings indicate that the operation of high-speed rail significantly contributes on economic growth. Although the introduction of high-speed rail does not markedly influence economic growth in medium-sized cities, it significantly contributes to the development of large cities, megacities, and supercities. Additionally, the impact becomes more pronounced as the city size increases. Heterogeneity analysis of urban industrial structure reveals that the opening of high-speed rail significantly boosts the economies of cities with a predominant focus on the secondary and tertiary industries, as compared to those reliant on the primary industry. The analysis of the transmission mechanism reveals that high-speed rail significantly contributes to economic growth by reducing carbon emissions and generating positive environmental effects. In addition, high-speed rail also drives economic growth by facilitating labor force agglomeration and technological innovation. Further research suggests that with the enhancement of transport infrastructure, regional economic growth tends to exhibit a trend of synergistic or convergent development.

The article is organized in the following manner: The second part describes the reality under study. The third part involves a mechanism analysis and research hypotheses. The fourth part covers variable selection and the construction of empirical models. The fifth part interprets the empirical results and conducts heterogeneity analysis. The sixth part involves an analysis of the transmission mechanism. The seventh part further discusses the findings, and the eighth part presents the conclusions and policy recommendations.

## 2. Characteristic facts

### 2.1. High-speed rail operation

Since the introduction of high-speed rail in 2008, the national operation mileage has reached 79,700 kilometers, with high-speed rail accounting for 700 kilometers, or 0.84% of the total. As of the end of 2022, the national railway operation mileage has expanded to 155,000 kilometers, with high-speed rail covering 42,000 kilometers, making up 27.1%. By the end of 2023, the national railway operation mileage reach approximately 158,000 kilometers, with high-speed rail operation mileage accounting for around 44,500 kilometers.

The Yangtze River Delta region, known for its vibrancy, openness, and innovation, plays a crucial role in paving the way for regional integration. The development of transportation infrastructure stands as a pioneering sector, providing essential support and serving as a vital catalyst for the high-quality growth of the Yangtze River Delta integration. In the past five years, remarkable progress has been achieved in rail transportation within the Yangtze River Delta region. The railway area density has increased from 294 kilometers per 10,000 square kilometers at the end of 2017 to 392 kilometers per 10,000 square kilometers at the end of 2022. Similarly, the highway area density increased from 413 kilometers per 10,000 square kilometers at the end of 2017 to 466 kilometers per 10,000 square kilometers at the end of 2022. The high-speed railway network has expanded to cover a total length of 6,668 kilometers, doubling since the end of 2017. The Yangtze River Delta boasts a cutting-edge high-speed rail network that is safe, efficient, and well-integrated. The region’s multi-level urban rail transit system and high-speed expressway network effectively cater to the high-frequency connectivity demands of urban agglomerations.

### 2.2. Economic growth in the Yangtze River Delta region

The Yangtze River Delta region, encompassing Shanghai Municipality, Jiangsu Province, Zhejiang Province, and Anhui Province, stands as one of China’s most populous and highly urbanized urban clusters. This thriving region accounts for a significant one-fourth of the country’s total economic output. Over the period from 2014 to 2022, the total economic output of the YRD has shown a steady upward trend. In 2022, the region’s GDP reached an impressive 29.03 trillion yuan, accounting for approximately 24% of the national total and playing a pivotal role in driving the economic growth of China. The average rate of GDP growth in the Yangtze River Delta region stood at 4.87% in 2022. Over the years, the region’s economic growth rate has experienced a gradual deceleration, particularly in 2020 when it reached its lowest level in recent times. This slowdown can be largely attributed to the outbreak of the pandemic, which significantly influenced the region’s economic performance.

**Fig 1:**
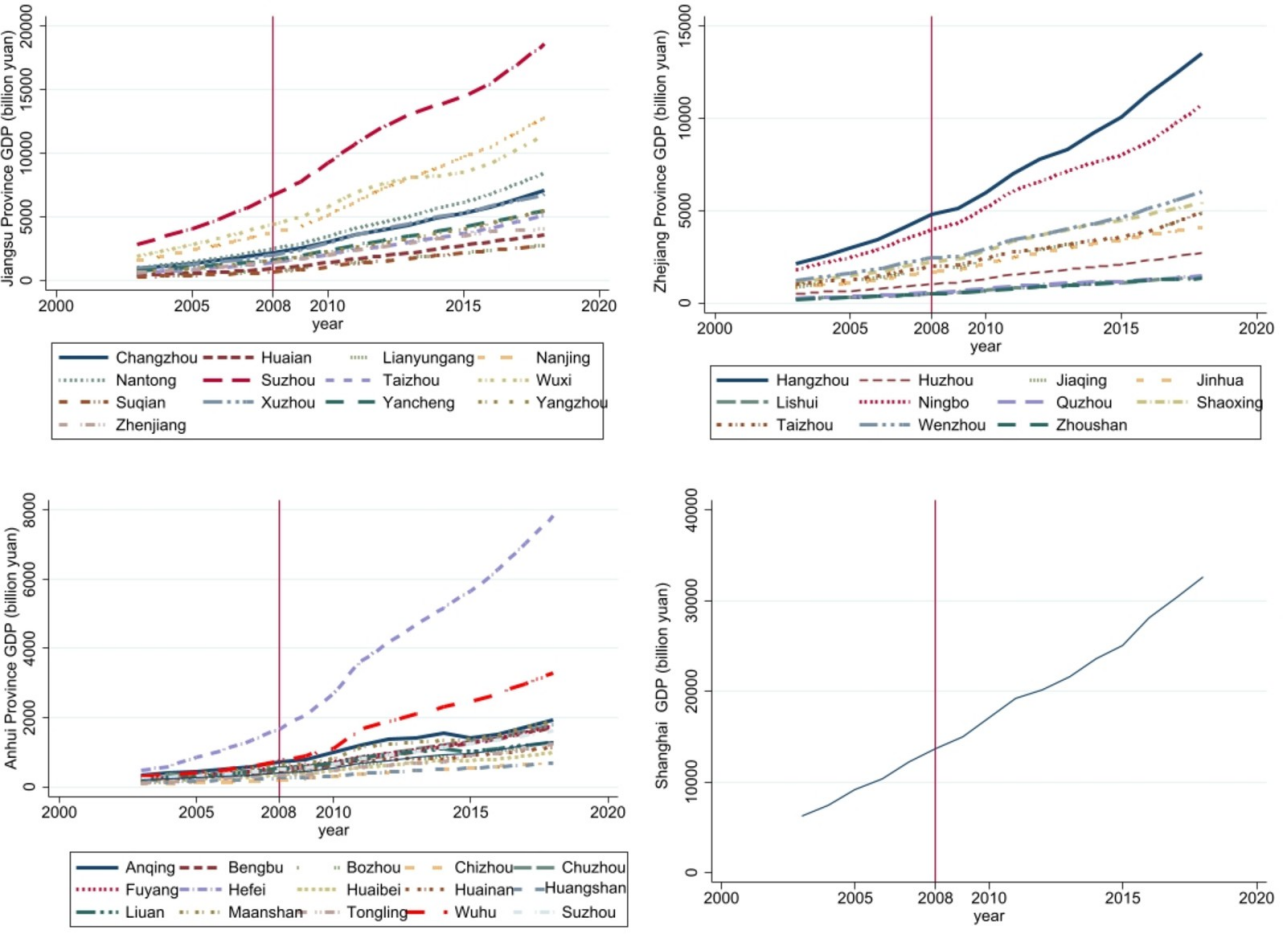
The economic growth trends of different regions in the Yangtze River Delta from 2003 to 2020. (a) The economic growth trend of Jiangsu Province; (b) The economic growth trend of Anhui Province; (c) The economic growth trend of Zhejiang Province; (d) The economic growth trend of Shanghai.

Fig 1 depicts the economic growth trends of cities in the Yangtze River Delta region before and after the introduction of high-speed rail. As is evident from the graph, before the introduction of high-speed rail, there was a relatively minor disparity in economic growth among cities. However, since the commencement of high-speed rail operations in 2008, the economic disparities between cities in the Yangtze River Delta have gradually widened.

## 3. Literature review

Due to the widespread construction of high-speed rail networks globally, researchers extensively focused on the economic and social effects triggered by high-speed rail operations. For example, Komikado et al. [17] investigated the relationship between the accessibility of high-speed rail in Japan and regional innovation. They found that the presence of high-speed rail stations significantly boosted regional knowledge productivity, and the promotional effect of high-speed rail on regional innovation primarily depended on opportunities for employee interaction. Miwa et al. [18] regarded the opening of high-speed rail in Japan as a quasi-experiment and discovered a significant positive impact of high-speed rail development on regional innovation. This was attributed to the improved accessibility provided by high-speed rail, which may have generated more opportunities for knowledge exchange among human capital. Cascetta et al. [19] conducted an analysis of the impact of high-speed rail operations in Italy a decade later on economic growth, accessibility, and regional equity. The study unveiled that high-speed rail boosted the contribution rate to transportation accessibility in the regions along the route by 32%, resulting in an extra 2.6% growth in per capita GDP and a decrease in regional inequality. In the case study of the Milan-Bologna high-speed rail corridor in northern Italy, Matteo et al. [20] assessed the impact of transportation infrastructure on economic performance. The study indicated that high-speed rail was effective for both central cities and secondary zones, but its impact varied depending on the unique characteristics of the location.

### 3.1. Study on the economic growth impact of high-speed rail

China serves as the most common case study for researching the economic impact of high-speed rail. While most studies consider high-speed rail to be a significant factor in promoting economic growth, there is still no consensus on the extent of its economic impact. High-speed rail improved economic quality and development levels by enhancing accessibility, accelerating factor mobility, optimizing industrial structure, reducing environmental pollution, and optimizing income distribution [21]. Liu and Zhang [22] analyzed the economic effects of high-speed rail from the perspectives of travel time, accessibility, and economic productivity. The high-speed rail not only improved travel time between cities but also contributed approximately 25–45% to accessibility. In terms of economic productivity, the elasticity of high-speed rail on per capita regional GDP was 0.28. Yao, Fang, and He [23] provided evidence that high-speed rail in China could help avoid the middle-income trap. The time-space compression effect resulting from high-speed rail exhibited a 0.6% elasticity on economic growth. The effective boundary range for the impact was 200–1200 kilometers for provincial-level cities and 50–300 kilometers for prefecture-level cities.

Some scholars argue that high-speed rail has a negative impact on China’s economic growth. Qin’s [24] study provided empirical evidence for the potential reshaping of economic activities through transportation infrastructure investment. The research found that after the speed upgrade of the railway, the GDP and per capita GDP of the counties along the route decreased. Jia et al. [25] argued that a city should adjust and reorganize its local resources to benefit from high-speed rail. Otherwise, the introduction of high-speed rail is likely to have a negative impact on local economic growth, potentially turning it into a peripheral city. Liang et al. [26] studied the economic effects of high-speed rail on underdeveloped regions using the Guangdong-Guangxi-Guizhou high-speed rail as an example. They found that in the short term, this high-speed rail did not significantly promote economic growth in the areas along its route, indicating a need for longer-term observation to assess changes in regional economic growth.

### 3.2. The impact of the HSR on the unbalanced regional economic growth

The high-speed rail appears to bring numerous benefits to cities, but some scholars express concerns about the significant costs and potential disruption to the existing urban structure, as well as the balanced development between cities. Jiao et al. [27] assessed the locational endowment features brought about by high-speed rail development by constructing indicators of high-speed rail accessibility and connectivity. They concluded that high-speed rail development resulted in uneven economic growth across cities with different geographical regions and population sizes. Jin et al. [28] argued that high-speed rail may have exacerbated China’s economic disparities. Specifically, they found that high-speed rail made a significant contribution to the economic growth of large cities with insignificant spillover effects. However, the impact of high-speed rail on the economic growth of small and medium-sized cities was not significant, with spillover effects being significantly negative. Wang et al. [29] contended that high-speed rail does not always bring growth opportunities. They argued that high-speed rail connections led to severe population loss and economic decline in small and medium-sized cities, further exacerbating regional disparities. Wang and Zhang [30] argued that caution should be exercised when constructing new high-speed rail stations in small and medium-sized cities. They emphasized the need to evaluate the economic feasibility of establishing new high-speed rail cities and the adverse effects of low urban expansion efficiency.

Some scholars argue that the development of high-speed rail can effectively promote the convergence of the Chinese economy. According to Chen & Haynes [31], the study indicated that since the development of high-speed rail (HSR), regional economic disparity has decreased, suggesting that HSR has facilitated economic convergence across regions in China. Zhang et al. [32] found that the opening of high-speed rail (HSR) had a positive and significant impact on provincial economic equity. However, with the expansion of the high-speed rail network, this positive effect gradually diminished. Huang [33] discovered that high-speed rail (HSR) may have reduced regional disparities in daily accessibility while widening the gaps in locational accessibility and potential accessibility. HSR indirectly enhances regional economic growth by improving spatial connections between cities and promoting regional integration.

### 3.3. High-speed rail research using nighttime light data

Other literature related to this paper includes scholars examining the economic effects of high-speed rail using nighttime light data. Nighttime light is considered an effective indicator for measuring urban land expansion [34, 35], population size [36], and urban economic development [37]. However, most scholars only use traditional DMSP-OLS and NPP-VIIRS nighttime light data as a single measure of urban economic activities. Deng et al. [38] utilized DMSP/OLS nighttime light brightness to assess the actual development status of 124 high-speed rail stations in China and delved into the reasons behind the diverse development patterns of high-speed rails. Guo et al. [39] examined the impact of the Beijing-Guangzhou high-speed rail line on urban economic development using DMSP/OLS and VIIRS nighttime light data from 2002 to 2018. Liang et al. [26] replaced the economic growth levels in the high-speed rail corridor areas with NPP-VIIRS remote sensing data from 2012 to 2017 and studied the effectiveness of high-speed rail on regional economic growth in underdeveloped areas. Wang et al. [40] utilized DMSP/OLS nighttime light data from 2004 to 2013 as a measure of urban economic activity. They argued that high-speed rail requires time to accumulate before exerting economic effects and did not show significant promotional impacts on cities along the line in the short term. Dong et al. [41] utilized satellite images and government online documents to identify 180 new high-speed rail cities. They investigated the underlying reasons for the economic growth stimulated by newly built high-speed rail stations and found that the key factors distinguishing "ghost towns" from thriving small towns were their locations and access to markets. Niu et al. [42] analyzed the land use effects of high-speed rail stations using nighttime light data and found that the opening of high-speed rail led to an increase of approximately 4.4% in urban land use intensity. Wang and Zhang [30] utilized nighttime light data to assess the distribution of urban economic activities and investigated the reshaping effect of newly built high-speed rail stations on urban economic activities. The study found that after the construction of new high-speed rail stations, the gravitational core of urban economic activities did not significantly shift towards the station areas.

### 3.4. Research limitations and contributions

While a significant number of researchers acknowledge the crucial importance of high-speed rail for national economic development, there are still the following shortcomings: (1) Currently, there is still a lack of research on the economic effects of high-speed rail based on micro-level data. Although nighttime light data is frequently used as a proxy for some economic indicators (such as population and economic growth) in analyzing the impact of high-speed rail on urban economies, it does indeed reflect the economic influence of high-speed rail to some extent, but these indicators are singular and one-sided. This implies that relying solely on one aspect of indicators may lead to divergent or even contradictory conclusions, resulting in a one-sided and inaccurate presentation of research findings. (2) Most studies analyze the short-term changes in socio-economic factors following the operation of high-speed rail, with fewer focusing on the medium- to long-term impacts of high-speed rail on economic growth. Current research lacks continuous observation and analysis over longer time series. (3) The controversy surrounding the impact of high-speed rail on economic growth and regional balance necessitates further empirical quantitative analysis, especially across various types and scales of cities.

Therefore, the main research contribution of this study is: (1) This study introduces a new data source, namely, new nighttime light data, distinct from traditional nighttime light data (NPP/VIIRS and DMSP/OLS). This dataset addresses the issue of the inability to simultaneously use DMSP-OLS and NPP-VIIRS nighttime light data, while also extending the time span covered by conventional nighttime light data. This ensures the continuity and availability of the research. Additionally, this study combines new nighttime light data with traditional economic indicators (GDP) to comprehensively measure urban economic activities, overcoming the challenge of a single indicator’s inability to accurately and comprehensively assess economic levels. (2) Due to the time required for large-scale transportation infrastructure to accumulate its effects, this study tracks the impact of high-speed rail on economic activities annually following its opening, including the effects over ten years of high-speed rail operation. This effectively demonstrates the medium to long-term effects of high-speed rail on the economy. (3) In comparison to previous studies, this paper offers abundant empirical evidence regarding the controversies surrounding the impacts of high-speed rail on economic growth and economic disparities. It includes analyses of urban scale, urban industrial structure, high-speed rail cities versus non-high-speed rail cities, as well as regional balance.

## 4. Variable selection and empirical model

### 4.1. Variable selection and data sources

#### 4.1.1. Economic activity: New nighttime light data

In developing countries, the methods and statistical systems for estimating GDP are generally less advanced, and the government’s statistical infrastructure is often inadequate, leading to relatively low quality of GDP data. The statistical methods utilized for China’s GDP data are somewhat outdated, and within the traditional framework of local performance assessment, local governments tend to inflate GDP statistics. Economists are thus seeking a more objective indicator to replace GDP or to supplement its shortcomings in statistical accounting. Nighttime light data proves particularly valuable for assessing the socio-economic dynamics of countries and regions where reliable official statistics may be lacking. Chen & Nordhaus [43] demonstrated that DMSP data served as a valuable supplement to current economic indicators, particularly for regions with poor or missing data quality. Henderson et al. [44] found that DMSP nighttime light data could serve as a useful proxy variable for GDP growth, capable of tracking both long-term and short-term economic fluctuations. Xu et al. [45] calculated the actual economic growth of China and its provinces using nighttime light data and official GDP statistics. The results revealed a significant positive correlation between light intensity and GDP under different estimation methods. Additionally, the average actual economic growth rate from 1993 to 2012 did not align with official data, showing a nationwide discrepancy of 1.02 percentage points lower. However, doubts have always existed regarding the effectiveness of using DMSP data as proxies for economic variables and measuring the level and changes of economic activity. Gibson et al. [46, 47]called for the recognition that the DMSP data was not intended to assist economists, and many economists excessively relied on DMSP data, leading to improper usage. The DMSP data suffered from flaws such as blurriness, top coding, and lack of calibration. Economists should have turned to utilizing VIIRS nighttime light data. Pérez-Sindín et al. [48] tested the suitability of nighttime light data for estimating Regional Domestic Product (RDP) in various urbanized areas of Colombia. The study results indicated that all nighttime light data sources (DMSP, VIIRS, DMSP/VIIRS composite) served as good indicators for the municipal RDP model, with VIIRS data demonstrating the best fit. Hu and Yao [49] argued that GDP growth in national accounting was not sufficiently accurate for low- and middle-income countries, while nighttime light data played a significant role in enhancing GDP measurement. Yu et al. [50] utilized VIIRS nighttime light data to assess the accuracy of econometric models used for correcting per capita GDP growth. The results indicated that the GDP estimation model could reduce artificial biases in simulated per capita GDP growth rates, with an average accuracy of 89% on simulated data.

Compared to GDP, nighttime light data possesses the following advantages. Firstly, nighttime light data is not influenced by selection biases in micro household surveys. Light data remains unaffected by human factors, thereby minimizing the possibility of falsification and offering a more objective alternative to GDP statistics that can be influenced by human behavior in specific environments. Furthermore, the level of market integration and price consistency among different regions within developing countries is lower, making the calculation of nominal GDP a challenging task. Nighttime light data remains unaffected by data manipulation or changes in statistical standards during macroeconomic account revisions. Lastly, in many developing countries, a significant portion of economic activities occurs outside the formal sector and thus are not included in GDP calculations. The advantage of using nighttime light data to measure economic activities lies in its ability to capture informal activities, with data being almost real-time and acquisition costs being low.

Currently, there are two main sources of nighttime light data, namely the Defense Meteorological Satellite Program’s Operational Linescan System (DMSP-OLS) and the Visible Infrared Imaging Radiometer Suite (VIIRS) nighttime light data from the Suomi National Polar-orbiting Partnership (NPP). Although both types of datasets are considered good substitutes for detecting population dynamics and socio-economic activities, their application is always constrained by the quality and availability of time spans [16]. For example, the DMSP-OLS data ceased production in 2013, rendering it increasingly outdated. Furthermore, the DMSP data suffers from issues such as lack of orbital radiance calibration, saturation problems, and blurring, greatly limiting its potential applications. While NPP-VIIRS data boasts good quality and superior detection capabilities, its available time span is relatively short, making it unsuitable for long-term analysis. Therefore, the "NPP-VIIRS-like" nighttime light dataset spanning from 2000 to 2018 produced by Chen et al. [16], referred to as the "new nighttime light dataset," serves as a valuable data source for this study. This dataset possesses quality similar to NPP-VIIRS nighttime light data, addressing the issue of the inability to simultaneously utilize both DMSP-OLS and NPP-VIIRS nighttime light datasets and extending the available time span of traditional nighttime light data. To assess the effectiveness of this dataset, Chen et al. [16] selected six countries (the United States, Italy, China, Brazil, South Africa, and Australia) as samples and found that at a regional scale, all countries demonstrate acceptable accuracy. This indicates that the "new nighttime light dataset" possesses good quality with spatial variations.

The new nighttime light dataset for the years 2000 to 2018 is available for free access via the Harvard Dataverse platform. The data is stored in GeoTIFF format, with specific parameters outlined as follows. The details are shown in Table 1.

**Table 1:** Specific Parameters of the "new nighttime light data set".

Parameters	Value
Coverage Area	Global
Time Span	2000–2018
Spatial Coordinates	WGS84
Unit	nWcm-2sr-1
Spatial Resolution	15 arc-seconds
Product Cycle	Year

Certainly, using nighttime light data to measure the economic growth of a country or region is not entirely accurate. However, this does not hinder its beneficial role as a supplementary traditional economic indicator from an academic perspective. Although nighttime light data is not a perfect measure of economic activity, as long as its measurement error is unrelated to the errors in official statistics, the index derived from combining official data with nighttime light data is bound to be a statistically more accurate measure of economic activity [50]. Therefore, this study combines the "new nighttime light dataset" with regional GDP as dual indicators to measure economic activities to comprehensively assess the economic growth levels of various cities in the Yangtze River Delta. The GDP data is sourced from the China City Statistical Yearbook.

### 4.1.2. Independent variable: Operation of high-speed rail

The opening times of high-speed rail lines and the overall count of high-speed rail connections between cities are obtained from official documents of the National Railway Administration of the People’s Republic of China, the "China Railway Yearbook," the website of China Railway Corporation, and information manually compiled and cross-checked with data from the China Railway Corporation’s 12306 website and the "Qunar" travel website.

#### 4.1.3. Control variables

The economic activity of cities can be more accurately measured using nighttime light data, which offers a higher level of reliability. However, this data may not provide detailed information about specific economic activities. To account for the internal economic characteristics of each city, we introduce economic factors, social factors, and urban features of the cities as controlled variables. Furthermore, we also incorporate the variables of highways, waterways, and civil aviation to assess the level of urban transportation infrastructure, as illustrated in Table 2. Table 3 presents descriptive statistics of nighttime light brightness, high-speed rail operations, and controlled variables.

**Table 2:** Selected indicators for controlled variables.

Controlled Variables	Indicator	Description
Fixed asset investment	Fixed	Fixed asset investment as a percentage of GDP (%)
Degree of openness	Open	The proportion of actual utilized foreign investment in GDP (%)
Average wage	Wage	Average worker’s wage/salary (yuan/RMB)
Fiscal expenditure	Exp	The proportion of urban government’s budgetary expenditure to GDP (%)
Educational level	Teacher	Number of full-time teachers in higher education per ten thousand people (person)
Highway	Highway	Highway passenger traffic volume (10,000 persons)
Waterway	Water	The waterborne passenger volume (ten thousand people)
Civil aviation	Avi	Civil aviation passenger volume (ten thousand people).
Regional land area	Area	Urban administrative area land area (in square kilometers)

**Table 3:** Descriptive statistics of variables.

Variables	Mean Value	Standard Deviation	Minimum Value	Maximum Value
DNnew	1.343	1.931	0.015	12.646
GDP	2680.169	3830.291	75.503	32679.87
HSR	0.343	0.475	0	1
Fixed	64.42	23.00	19.357	146.875
Open	0.463	0.320	0.023	2.429
Wage	42760.03	22981.92	7992.76	142983
Exp	13.989	7.990	4.910	148.916
Teacher	5476.044	9334.62	87	52531
Highway	10456.54	9166.448	1168	68895
Water	146.044	386.837	0.57	2922
Avi	254.963	871.413	0	9412
Area	8519.535	4227.417	1113	18399

### 4.2. Endogenous issues of high-speed rail stations

The allocation of high-speed rail stations is not randomly assigned to cities but rather closely tied to the population size and economic development level of those cities. Hence, addressing the endogeneity of high-speed rail stations becomes a crucial concern. This article utilizes the "minimum cost path method" introduced by Büchel & Kyburz [51] to create instrumental variables for the high-speed rail stations. The approach is divided into three main steps, as follows. The first step involves selecting a node city based on a comprehensive evaluation of transportation’s strategic importance and the population size. Next, the "minimum cost path" feature in ArcGIS is utilized to draw the minimum cost path using a 250 x 250meter grid. Finally, an index *LCP*_*i*_ is generated based on the intersection of the minimum cost path with the city boundaries. If a minimum cost path passes through a city, *LCP*_*i*_ is set to 1; if all minimum cost paths bypass the city, *LCP*_*i*_ is set to 0. Nonetheless, given that the LCP represents cross-sectional data unaltered by time, we introduce an interaction term between the LCP and a time dummy variable, serving as an instrumental variable *LCP*_*it*_.

### 4.3. Establishment of empirical model

In this study, the opening of high-speed rail is treated as a quasi-natural experiment. Cities that have high-speed rail stations along the route are considered the treatment group, while cities without high-speed rail serve as the control group. Due to the endogeneity issue of high-speed rail stations, the empirical model will be conducted in two stages. In the first stage, instrumental variable (IV) estimation is employed using the *LCP*_*it*_ (minimum cost path) as an instrument to estimate the variable for the opening of high-speed rail. The second stage equation examines the impact of high-speed rail operation on economic growth.


$$HSR_{i,t}=\alpha_{1}+\beta_{1}LCP_{it}+\varphi \sum X_{it}+\phi_{t}+\lambda_{i}+\varepsilon_{it}$$ (1)

$$Y_{i,t}=\alpha_{2}+\beta_{2}{HSR}_{it}+\chi \sum X_{it}+\pi_{t}+\sigma_{i}+\mu_{it}$$ (2)

Y_it_ represents the economic growth levels of each city in the Yangtze River Delta region, measured using nighttime light intensity (*DN*new_it_) and *GDP*_it_. The binary variable *HSR*_*i*,*t*_ indicates whether a city launched HSR service during that year, and *LCP*_*it*_ is the instrumental variable for *HSR*_*i*,*t*_. The coefficient *β*_2_ captures the "treatment effect" of public policy identified by the double difference in cross-sectional and time-series caused by the opening of high-speed rail. *X*_*it*_ represents control variables, including average wage level, fixed asset investment, degree of openness, and city size, which measure internal characteristics of the cities. *π*_*t*_, *ϕ*_*t*_ and *σ*_*i*_, *λ*_*i*_ represent time and regional effects, respectively. *ε*_*it*_, *μ*_*it*_ represents temporary shocks.

## 5. Analysis of regression results

### 5.1. Baseline regression results analysis

The regression results are presented in Table 4. Columns (1) and (2) present regression results for measuring economic growth using nighttime light and GDP, respectively, without addressing the endogeneity of high-speed rail stations. Column (3) shows the results of the instrumental variable model, where there is a highly significant coefficient between the instrumental variable and the high-speed rail opening variable. When employing night-time light data as a measure of economic activity, the F-statistic of the first-stage regression equals 28.1904, which is greater than 10. This leads to the rejection of the null hypothesis H0 that weak instrumental variables exist. When GDP is used as a gauge for economic growth, the F-statistic of the first-stage regression equals 27.537, also greater than 10, thus rejecting the initial hypothesis. This signifies that the instrumental variable has successfully passed the weak instrumental variable test. Columns (4) and (5) present regression results after addressing the endogeneity of high-speed rail stations. The results indicate that regardless of using nighttime light or GDP to measure economic activity, the opening of high-speed rail significantly promotes economic growth in cities along the rail line. Column (4) and column(5) indicate that the opening of high-speed rail significantly enhances economic growth in the cities along the high-speed rail route, and the coefficient of promotion is higher compared to the case where the endogeneity of high-speed rail is not considered. This suggests that neglecting the endogeneity of high-speed rail would lead to a significant underestimation of the regression results. The reason for the Instrumental Variable (IV) estimation result being tenfold larger than the standard Difference-in-Differences (DID) estimation can be traced back to three factors. Firstly, the instrumental variable estimation reflects the local effect of a sub-sample that exhibits higher economic growth. Secondly, even a slight deviation from the exclusion restriction due to weak instrumental variables can magnify the estimation bias. Lastly, the baseline regression might contain omitted variables that result in the estimates converging towards zero. We’ve ruled out the second factor within the manuscript, and while the first and third factors may be present, they do not fundamentally impact our conclusions.

**Table 4:** Regression results of high-speed rail operation on economic growth.

	(1)	(2)	(3)	(4)	(5)
Dependent Variable	DNnew	GDP	HSR	DNnew	GDP
Model	Time-varing DID		IV	Time-varing DID&IV	Time-varing DID&IV
HSR	0.125	56.46		1.193***	2,305***
	(0.077)	(115.8)		(0.389)	(791.7)
LCP			0.084**		
			(0.039)		
Constant	1.159*	973.9	-0.359***	1.321**	1,158
	(0.646)	(1,667)	(0.088)	(0.633)	(1,659)
Observations	651	659	659	651	659
R-squared	0.424	0.6586	0.470	0.428	0.6632
Control Variables	YES	YES	YES	YES	YES
Individual Fixed Effects	YES	YES	YES	YES	YES
Time Fixed Effects	YES	YES	YES	YES	YES

Note:

***, **, and * show estimated coefficients that are significant at the 1%, 5%, and 10% confidence levels, respectively. Robust standard errors for coefficients clustered at the city-year level are in parentheses.

### 5.2 Parallel trend test

In order to analyze the dynamic relationship between high-speed rail operation and economic growth, we incorporated a set of dummy variables in the standard regression model to track the annual impact of high-speed rail operation on urban economic activity within the Yangtze River Delta region.

$$Y_{it}=C_{0}+\partial_{1}hsr_{it}^{-3}+\partial_{2}hsr_{it}^{-2}+\ldots +\partial_{10}hsr_{it}^{+10}+\omega \sum X_{it}+\tau_{t}+\rho_{i}+\varepsilon_{it}$$ (3)

Y_*it*_ represents nighttime light (*DNnew*_it_) and *GDP*_it_. The superscript of *hsr*_*it*_ represents the relative time value obtained by subtracting the year of high-speed rail opening in a city from the current year. Time values before 4 periods prior are merged into the fourth period. For cities that did not have high-speed rail throughout the study period, all dummy variables are set to 0, and the baseline reference group is the first period.

**Fig 2:**
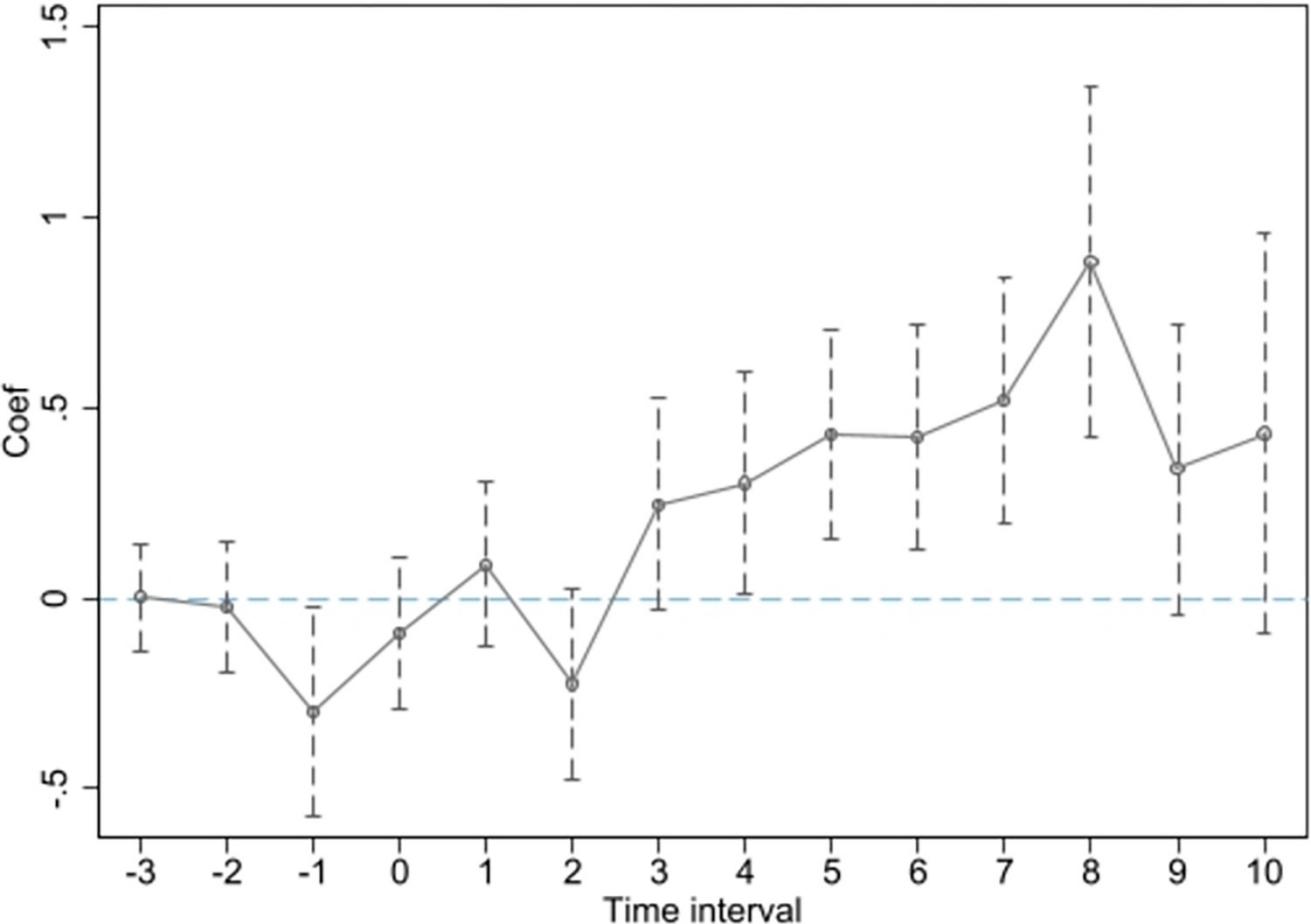
The parallel trend test conducted using nighttime light data.

**Fig 3:**
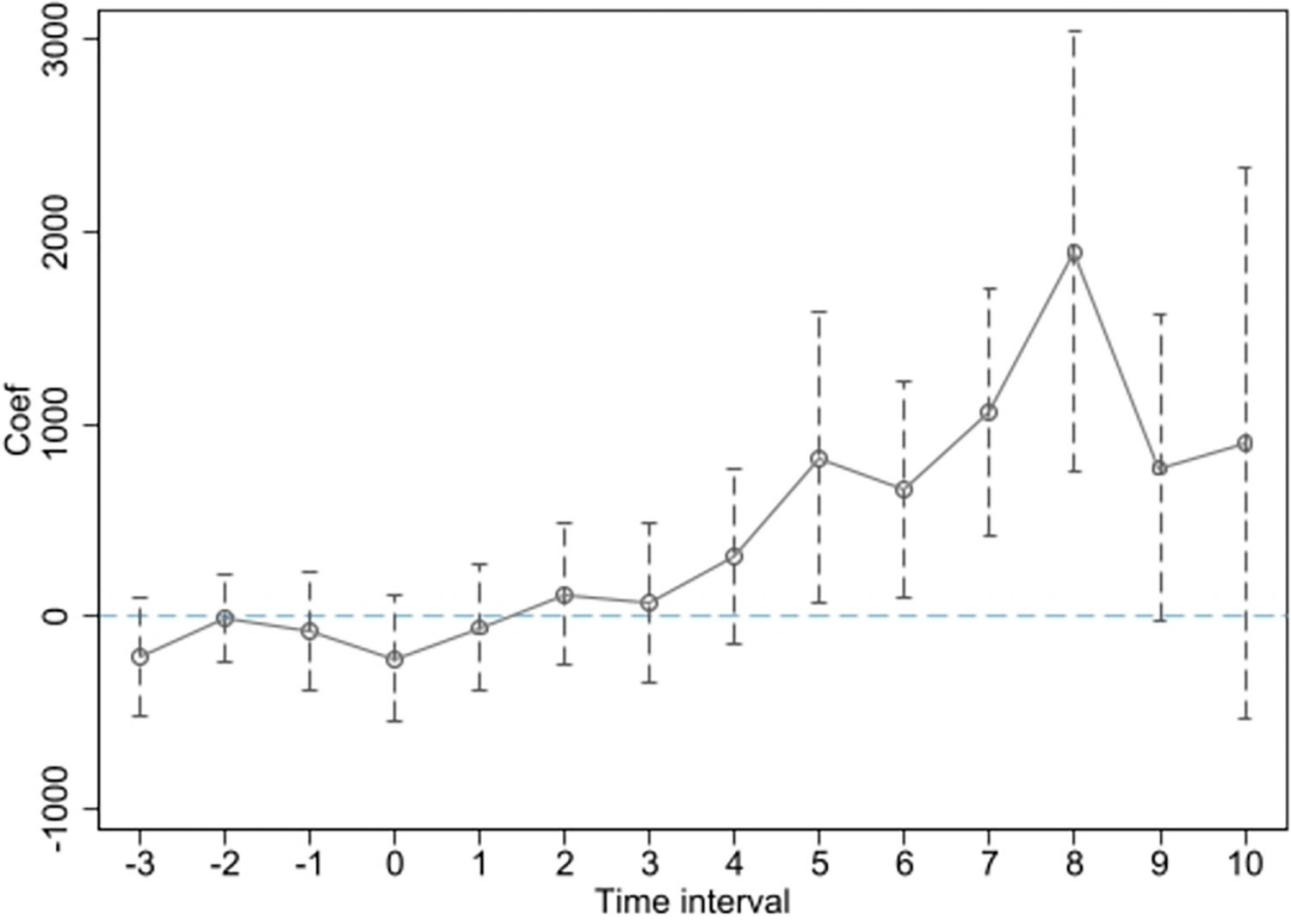
The parallel trend test conducted using GDP.

Figs 2 and 3 demonstrate a high degree of consistency, indicating that prior to the opening of high-speed rail, there is no significant difference in nighttime light intensity or GDP between cities, thereby validating the parallel trends assumption. It is worth noting that Fig 2 passes the significance test two years after the opening of the high-speed rail, while Fig 3 passes the significance test four years after the opening. This provides evidence for the potential lagged effect of transportation infrastructure expansion on regional economic growth. Furthermore, Figs 2 and 3 indicate that over time, the positive impact of high-speed rail on economic growth increases. This suggests that the economic effects of large transportation infrastructure projects become more significant as time accumulates.

### 5.3 Robustness test

#### 5.3.1 Transforming independent variables, reselecting samples, and propensity score matching (PSM)

In order to assess the robustness of the baseline regression results, this study re-conducted the regression by employing the overall count of high-speed rail routes reaching the destination city as an alternative measure of high-speed rail operation. Secondly, during the initial phase of transportation infrastructure development, central cities such as Shanghai, Hangzhou, Hefei, Nanjing, etc. were the first to be connected. These central cities had an initial advantage in economic development compared to other cities, resulting in relatively high nighttime light brightness in these areas. To alleviate the endogeneity issue caused by this situation, this study excluded the central cities from the analysis and re-conducted the regression. Moreover, the challenge of "quasi-natural experiments" used for policy evaluation is that the subjects may not be randomly assigned to the experimental and control groups. Given the substantial differences between cities, it is essential to employ propensity score matching (PSM) before regression analysis. PSM aims to enhance the resemblance between observed data and randomized experimental data by matching and resampling. It selects control group cities that exhibit comparable characteristics to the treatment group, thereby reducing selection bias to the greatest extent possible.

The findings, as displayed in Table 5, remain robust even when adjusting the independent variables, excluding central cities, or employing PSM. Cities with high-speed rail connections exhibit a higher level of nighttime light intensity in comparison to cities lacking high-speed rail service, with an increase ranging from 0.167 to 1.567 units. A similar situation is observed with GDP, where cities with high-speed rail passing through have a GDP higher by 267.1 to 3448 billion yuan compared to non-high-speed rail cities. Furthermore, there exists a positive association between the number of high-speed rail lines opened in a city and its level of economic activity.

**Table 5:** The results after changing the independent variables, reselecting the sample, and conducting PSM.

Model	The number of high-speed rail lines		Reselecting the samples		PSM	
Explained Variable	DNnew	GDP	DNnew	GDP	DNnew	GDP
HSR			1.162***	2,584***	1.567***	3,448***
			(0.346)	(446.0)	(0.448)	(745.1)
HSRsum	0.167***	267.1***				
	(0.052)	(92.69)				
Constant	1.458**	1,336	1.448*	4,287***	0.862	2,736**
	(0.650)	(1,694)	(0.780)	(869.0)	(0.707)	(1,069)
Observations	651	659	588	596	487	495
Adjusted R2	0.440	0.6675	0.539	0.7728	0.364	0.5610
Control variables	YES	YES	YES	YES	YES	YES
City fixed effect	YES	YES	YES	YES	YES	YES
Time fixed effect	YES	YES	YES	YES	YES	YES

#### 5.3.2 The pre-effects and post-effects of high-speed rail on economic growth

The parallel trend test revealed a potential lag effect of high-speed rail operation on economic growth. Thus, this study explored the pre-effect and lag effect of high-speed rail implementation on economic growth by considering one period before operation, two periods before operation, one period after operation, and two periods after operation. As shown in Table 6, both nighttime light intensity and GDP yield a consistent conclusion: high-speed rail operations do not exhibit a leading effect on urban economic growth but show significant lag effects. This is congruent with the results of Na et al. [52], which suggest that the economic benefits of large transportation infrastructure take time to accumulate.

**Table 6:** The pre-effects and post-effects of high-speed rail on economic growth.

Model	one period before operation		two periods before operation		one period after operation		two periods after operation	
Explained Variable	DNnew	GDP	DNnew	GDP	DNnew	GDP	DNnew	GDP
F2.HSR	-0.264	-3.385						
	(0.328)	(375.7)						
F.HSR			0.218	748.8				
			(0.305)	(681.0)				
L.HSR					0.372	1,544**		
					(0.366)	(683.0)		
L2.HSR							0.973***	2,335***
							(0.308)	(779.1)
Constant	1.470**	2,428**	1.317**	1,981	1.339**	2,039	1.049	453.0
	(0.610)	(1,065)	(0.617)	(1,315)	(0.633)	(1,312)	(0.656)	(1,651)
Observations	566	572	605	612	605	612	566	572
Adjusted R2	0.375	0.7524	0.392	0.6449	0.391	0.6490	0.393	0.6365
Control variables	YES	YES	YES	YES	YES	YES	YES	YES
City fixed effect	YES	YES	YES	YES	YES	YES	YES	YES
Time fixed effect	YES	YES	YES	YES	YES	YES	YES	YES

#### 5.3.3 Placebo test

**Fig 4:**
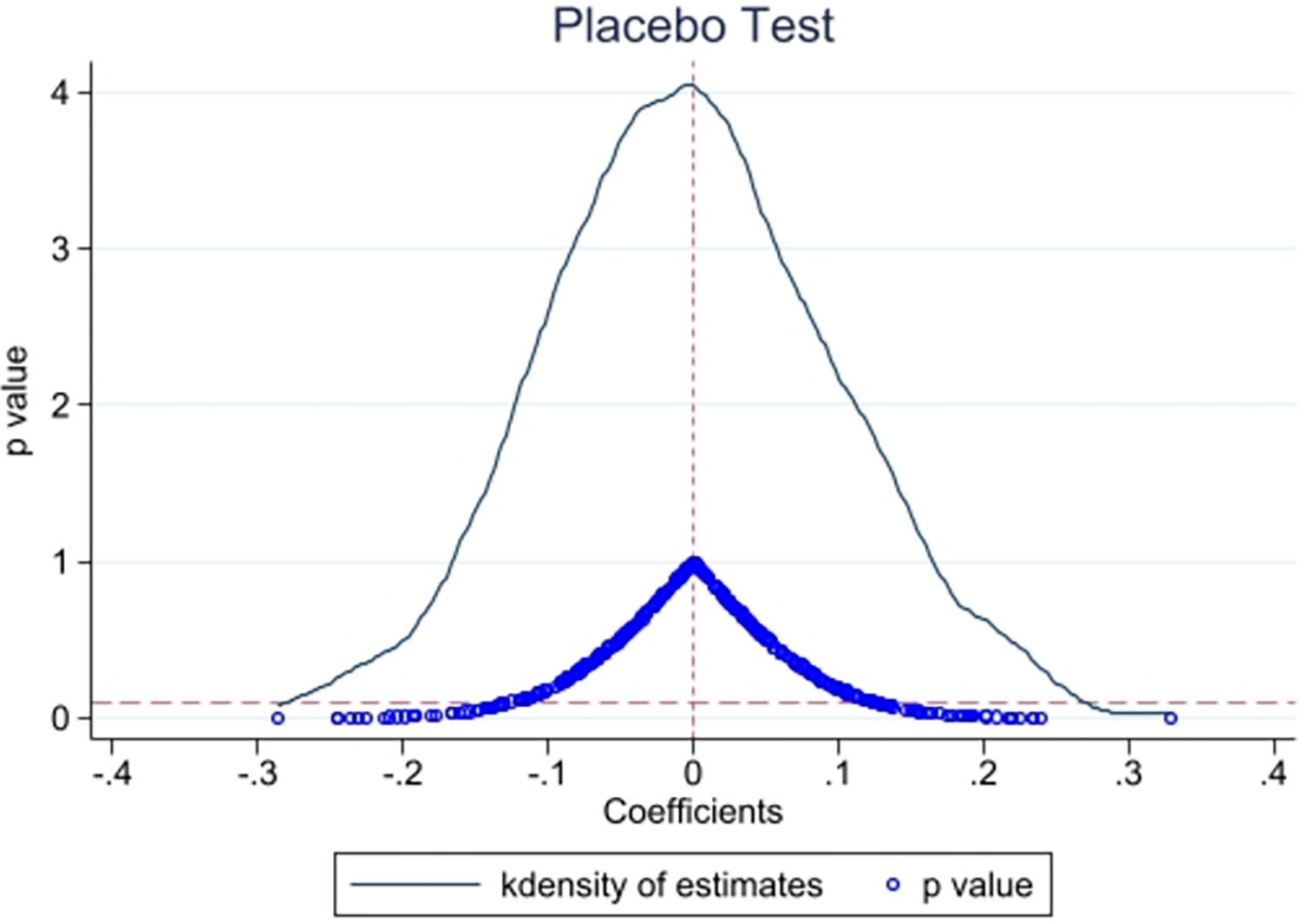
Placebo test.

After conducting 400 replications, the magnitude of the estimated coefficients is plotted on the x-axis, while the density value and the magnitude of the p-value of the estimated coefficients are plotted on the y-axis. As depicted in Fig 4, the estimated coefficients mainly cluster around the zero point, and the majority of estimates have a p-value greater than 0.1, indicating a lack of significance at the 10% level. These findings suggest that the regressions are robust and not predominantly influenced by external policies or random factors.

## 6. Heterogeneity analysis

### 6.1 Urban size

Following the notice issued by the State Council regarding "revising the standards for urban size classification," this article classifies the Yangtze River Delta region urban agglomeration into four categories: medium-sized cities (with an urban resident population of 500,000 to 1 million), large cities (with an urban resident population of 1 million to 5 million), mega-cities (with an urban resident population of 5 million to 10 million), and super-cities (with an urban resident population of over 10 million). Considering the substantial variations in internal characteristics among cities, the impacts of high-speed rail operations on economic growth differ across cities of different sizes. To examine the economic growth under various city characteristics, this study employs an interaction term between urban size and high-speed rail to perform heterogeneity analysis.

According to Table 7, when using nighttime light intensity as an indicator of economic growth, high-speed rail operations do not significantly affect the economic growth of medium-sized cities. However, they do significantly promote the economies of large, extra-large, and mega cities, with their effects becoming more pronounced as urban scale increases. When GDP is used as an indicator of urban economic growth, the results indicate significant promotional effects of high-speed rail on cities of various sizes. Moreover, the larger the city, the stronger the promotional effect observed. This phenomenon can be explained from the perspective of agglomeration economies in new economic geography [53, 54]. The reason is that the presence of external factors such as technology, resource allocation, and information exchange often leads to the concentration of enterprises or production activities. Due to the escalating economies of scale and the positive feedback mechanism, regions with agglomeration of production activities may have higher economic growth rates than other regions, leading to regional differences.

**Table 7:** Heterogeneity analysis.

Model	Urban size		Urban industrial structure	
Explained Variable	DNnew	GDP	DNnew	GDP
HSR* medium-sized cities	0.507	1,048*		
	(0.418)	(596.9)		
HSR* large cities	1.111***	1,554***		
	(0.378)	(565.7)		
HSR* mega-cities	1.448***	4,361***		
	(0.398)	(585.6)		
HSR* super-cities	4.316***	13,244***		
	(0.631)	(1,031)		
HSR* the secondary industry			1.253***	1,805**
			(0.432)	(792.1)
HSR* the tertiary industry			1.176***	2,101***
			(0.380)	(739.4)
Constant	-0.378	-4,550***	1.338**	1,099
	(0.533)	(880.3)	(0.640)	(1,663)
Observations	651	659	651	659
Adjusted R2	0.507	0.8685	0.428	0.6641
Control variables	YES	YES	YES	YES
City fixed effect	YES	YES	YES	YES
Time fixed effect	YES	YES	YES	YES

### 6.2 Urban industrial structure

The criteria for categorizing urban industrial structure is based on comparing the proportion of the three major industries in a city and assigning a value of 1 to the largest industry and 0 to the others. For instance, if the tertiary industry holds the largest proportion in a city, the industrial structure of that city is classified as being dominated by the tertiary industry.

According to Table 7, regardless of whether nighttime light or GDP is used as metrics for measuring economic growth, with the primary industry as the reference baseline, high-speed rail exhibits significant promotional effects on the economic growth of cities primarily focused on the secondary and tertiary industries. However, the impact coefficients show minimal differences between them. The reason for this may be attributed to the fact that the Yangtze River Delta region has a robust manufacturing base and is at the forefront of industrialization and informatization development in the country. For instance, the region has witnessed the formation of several internationally competitive innovation communities and industrial clusters in sectors such as integrated circuits, electronic information, and high-end equipment. In the new development pattern of dual circulation, the manufacturing industry in the Yangtze River Delta region holds a crucial position in the global manufacturing supply chain. The introduction of high-speed rail has greatly enhanced the export value of manufacturing enterprises by facilitating labor transfer and spatial technological spillovers. Conversely, the tertiary industry mainly caters to the consumption demands of the population, with a focus on promoting domestic circulation

## 7. Transmission mechanism analysis

There are three primary transmission channels through which high-speed rail promotes economic growth. The first channel is low-carbon transportation. High-speed rail serves as a prominent symbol of "green transportation" with its low-carbon emissions. This contributes to the shift towards green economic growth patterns and significantly drives sustainable economic development in the region. The second mechanism involves the enhancement of local labor supply and improvement of labor productivity through road infrastructure. Transportation infrastructure generates regional growth via agglomeration economies, with inflow of new workers being a typical model [55]. The third mechanism pertains to non-agglomerative knowledge channels. High-speed rail facilitates knowledge flow, leading to increased patent applications, even in cases where labor supply remains constant.

### 7.1 low-carbon

With traditional urban economies facing challenges to sustainable development, low-carbon transformation has emerged as a new economic growth model. Green and low-carbon initiatives are crucial for achieving sustainable growth. China is committed to enhancing its nationally determined contributions, adopting more potent policies and measures. It endeavors to reach peak carbon dioxide emissions by 2030 and strives for carbon neutrality by 2060. The introduction of these “dual carbon” goals is not only China’s national strategy in response to climate change but also an inevitable choice on the path to sustainable development. Especially under the “dual carbon” targets, China has adopted an unprecedentedly rigorous approach to environmental issues, including an intense crackdown on pollution.

To investigate whether high-speed rail operations reduce carbon emissions and have a positive impact on economic growth, we use new nighttime light data and GDP as the outcome variable and CO_2_ emissions as the mediating variable. The model is presented below:

$${lnCO}_{2,it}=\epsilon_{1}+\vartheta_{1}HSR_{it}+\sum \delta control_{it}+\kappa_{1t}+\phi_{1i}+\varepsilon_{it}$$ (4)

$$Y_{it}=\epsilon_{2}+\vartheta_{2}HSR_{it}+\lambda ln{CO}_{2,it}+\sum \varphi {control}_{it}+\kappa_{2t}+\phi_{2i}+\mu_{it}$$ (5)

Y_it_ represents the nighttime light intensity and GDP of city *i* in year *t*. *CO*_2,*it*_ represents the total carbon dioxide emissions of city *i* in year *t* (million tons). The data on carbon dioxide comes from the China Emission Accounts and Datasets (CEADs).

Table 8 presents the transmission mechanism of how high-speed rail (HSR) affects regional economic growth through low-carbon emission reduction. Column (1) shows that HSR operation can effectively reduce carbon emissions, while Columns (2) and (3) display a significantly positive HSR coefficient, albeit smaller than those in Columns (4) and (5) of Table 4. Moreover, the coefficient of CO2 is significantly negative, suggesting a mediating role of low-carbon emission in the impact of HSR operation on regional economic growth. This seems to contradict the traditional view of economic growth models, which associate strong correlation with carbon emissions due to the consumption of fossil fuels. However, the relationship between emission reduction and economic growth in China is no longer conflictual as it once was. Whether carbon reduction becomes a hindrance or a driving force for economic growth largely depends on the model of economic growth. If the business mindset and model remain in the traditional growth pattern established after the industrial revolution, characterized by high emissions and high resource consumption, high carbon emissions are undoubtedly a prerequisite for economic growth. However, if the model is a green transition, the technical characteristics of different resources vary, and the corresponding business models, organizational models, and institutional mechanisms will differ, potentially resulting in a mutually beneficial relationship between environmental and economic development. For example, traditional transportation mainly relies on high energy consumption. If emissions are reduced, fossil fuel use must decrease, leading to economic contractions. In contrast, China’s HSR represents a modern “green transport”. HSR minimizes carbon emissions and environmental pollution through energy-saving designs, low-carbon operations, waste processing, and environmental certifications. HSR employs electrification technology, with the national railway electrification rate currently at 74.9%. The annual fuel consumption of the national railway has decreased from a peak of 5.83 million tons in 1985 to the current 2.31 million tons, equivalent to reducing carbon dioxide emissions by 12.56 million tons annually. Data from the China State Railway Group Co., Ltd. shows that in terms of energy saving, the energy consumption per hundred kilometers per person of HSR is only 18% that of airplanes and about 50% that of large buses. Regarding land conservation, compared with four-lane highway, HSR occupies only 50% of the land, and the land occupied per unit of transportation is only 10% of that. In terms of environmental protection, the carbon dioxide emissions of HSR are only 6% of airplanes and 11% of cars. From 2012 to 2019, the increased passenger turnover of HSR, compared to the same passenger turnover completed by roads, reduced carbon dioxide emissions by 23.2 million tons. Thus, emission reduction could potentially drive the economy to leap from an old structure to a more competitive new structure, ushering in the transition to a whole new development paradigm.

**Table 8:** Analysis of the transmission mechanism based on low-carbon.

Model	(1)	(2)	(3)
Explained Variable	CO2	DNnew	GDP
HSR	-0.134**	1.086***	2,051***
	(0.0538)	(0.391)	(723.7)
lnCO2		-1.189**	-3,140**
		(0.475)	(1,249)
Constant	2.865***	5.498***	12,381***
	(0.0520)	(1.556)	(3,644)
Observations	571	571	571
Within R-sq.	0.3594	0.4318	0.7333
Control variables	YES	YES	YES
City fixed effect	YES	YES	YES
Time fixed effect	YES	YES	YES

Under the economic growth model of this new development paradigm, new demands and industries, such as electric vehicles and new energy in China, have unprecedented growth prospects. By converting carbon emissions into green new momentum through technological innovation, one can seize the commanding heights of green development, cultivate new green growth poles, and achieve a win-win situation for economic growth and carbon emissions. The development under the new economic growth model will inevitably shift from fossil fuel dependence to technology-driven innovation, realizing low resource consumption, less pollution and carbon dioxide emissions, and high-value outputs through technological innovation, thereby promoting the simultaneous advancement of low-carbon and economic growth.

### 7.2 Labor agglomeration

In the process of the emergence of agglomeration economies, drawing upon Marshall’s industrial scale theory, concerning transportation, three factors are closely interconnected: (1) agglomeration fosters the formation of enterprise clusters, leading to cost reductions in goods, materials, and services;(2) agglomeration generates a larger labor force, allowing for better matching of skills and improving productivity; (3) knowledge spillovers in agglomeration areas can promote productivity improvement [56]. Drawing on Graham [57], we utilize the concept of "effective density" as a measure to quantify the level of agglomeration. This measure essentially captures the regional clustering based on accessibility.


$${ED}_{i}^{t}=\frac{E_{i}^{t}}{\sqrt{SIZE_{i}}}+\sum_{j}^{i\neq j}\frac{\left|E_{j}^{t}\right|}{distane_{ij}^{t}}$$ (6)

*ED*_*it*_ represents the effective density of population in city *i* in year *t*, measured in ten thousand people per square kilometer. $E_{i}^{t}$ is the total employment in city *i*, *SIZE*_*i*_ is the administrative area of city *i*, and $E_{j}^{t}$ represents the total employment in city *j* in year *t*, ${distance}_{ij}^{t}$ represents the shortest distance of high-speed rail line between city i and city j in year t.


$${ED}_{it}=\epsilon_{3}+\vartheta_{3}HSR_{it}+\sum \partial control_{it}+\kappa_{3t}+\phi_{3i}+\varepsilon_{it}$$ (7)

$$Y_{it}=\epsilon_{4}+\vartheta_{4}HSR_{it}+\lambda {ED}_{it}+\sum \varphi {control}_{it}+\kappa_{2t}+\phi_{2i}+\mu_{it}$$ (8)

As presented in Table 9, column (1) shows that the operation of high-speed rail significantly enhances labor agglomeration. This is because transportation costs play a crucial role in determining the total amount of labor that firms can access. By improving high-speed rail infrastructure, larger-scale activities become more accessible by reducing travel time or travel costs, generating more positive agglomeration effects. On the contrary, regions with inefficient transportation infrastructure or limited accessibility may hinder the generation and distribution of agglomeration externalities. As shown in column (2) and column (3), labor agglomeration can significantly promote urban economic growth, highlighting the importance of human capital as a source of economic growth. This finding is consistent with the Uzawa-Lucas model, which utilizes human capital to explain the research approach to economic growth and emphasizes the crucial role of human capital externalities in explaining economic growth.

**Table 9:** Analysis of the transmission mechanism based on labor aggregation.

Model	(1)	(2)	(3)
Explained Variable	ED	DNnew	GDP
HSR	2.839*	0.907**	2,202***
	(1.465)	(0.361)	(813.4)
ED		0.101***	40.68**
		(0.0107)	(20.53)
Constant	9.289***	0.383	751.3
	(1.625)	(0.620)	(1,681)
Observations	651	651	651
Within R-sq.	0.1123	0.4994	0.6652
Control variables	YES	YES	YES
City fixed effect	YES	YES	YES
Time fixed effect	YES	YES	YES

### 7.3 Technology innovation

According to neoclassical growth theory, innovation resulting from technological progress is considered the most crucial component of sustainable economic growth. In line with this, the present study investigates how the operation of high-speed rail affects economic growth through technological innovation. The testing procedure is as follows: Step 1: Testing the existence of the mediating effect by examining whether high-speed rail operation influences technological innovation. Step 2: Including both high-speed rail operation and the mediating effect in the model to test the effectiveness of the mechanism. we have substituted the innovation level with the total count of approved patents for validation. The patent data, owing to its objectivity and timely updates, is extensively utilized in academic research.


$$Innovation_{it}=\alpha +\beta_{1}HSR_{it}+\sum \delta control_{it}+\kappa_{1t}+\phi_{1i}+\varepsilon_{it}$$ (9)

$$Y_{it}=\alpha +\beta_{12}HSR_{it}+\lambda Innovation_{it}+\sum \delta X_{it}+\kappa_{2t}+\phi_{2i}+\mu_{it}$$ (10)

Columns (1) to (3) in Table 10 depict the results of the intermediary effect test for technological innovation. The regression outcomes suggest a significant enhancement in innovation levels following the introduction of high-speed rail. As shown in columns (2) and (3), regardless of whether night-time lighting or GDP is used as a growth indicator, the coefficients of the double-difference terms diminish, and the regression coefficient of technological innovation is positively significant at the 1% level. This indicates that under the high-speed rail policy, technological innovation partially mediates urban economic growth. The above conclusions indicate that the operation of high-speed rail bolsters the overflow of knowledge and technology in cities, propels the concentration of innovative elements, fosters the elevation of city innovation levels, and positions technological innovation as a potent driver of urban economic development.

**Table 10:** Analysis of transmission mechanism based on technological innovation.

Model	(1)	(2)	(3)
Explained Variable	innovation	DNnew	GDP
HSR	3,581***	1.182***	2,249***
	(920.6)	(0.338)	(454.3)
innovation		0.000273***	0.807***
		(2.81e-05)	(0.0666)
		(1.13e-05)	(0.0278)
Constant	3,195**	1.738***	2,360***
	(1,430)	(0.464)	(724.4)
Observations	571	651	659
Within R-sq.	0.6128	0.5705	0.8928
Control variables	YES	YES	YES
City fixed effect	YES	YES	YES
Time fixed effect	YES	YES	YES

## 8. Discussion: Does high-speed rail operation lead to regional economic convergence or divergence?

As previously stated, there remains a debate within the academic sphere regarding whether high-speed rail contributes to regional convergence or divergence. While some researchers contend that high-speed rail widens economic disparities in China, others argue that its development effectively stimulates economic convergence. Consequently, the extent to which high-speed rail impacts China’s regional economic disparity remains unclear. This section, therefore, aims to address the question of whether high-speed rail operation promotes regional economic synergy or fosters divergence. Drawing on the methodology of Chen and Haynes [31], our study employs the coefficient of variation to quantify economic disparity within the Yangtze River Delta region.


$$CV_{w}=\frac{\sqrt{\sum_{i=1}^{n}\frac{p_{i}}{P}(x_{i}-\frac{1}{n}\sum_{i=1}^{n}x_{i}{)}^{2}}}{\frac{1}{n}\sum_{i=1}^{n}x_{i}}$$ (11)

In this context, *p*_*i*_ denotes the population of city i within the region, while P refers to the total population within the region. *x*_*i*_ signifies the GDP level (or the new nighttime light data DNnew) of region *i*, $\frac{1}{n}\sum_{i=1}^{n}x_{i}$ is the average level of GDP (DNnew) within the region. A larger *CV*_*w*_ value implies a greater disparity between regions.

**Fig 5:**
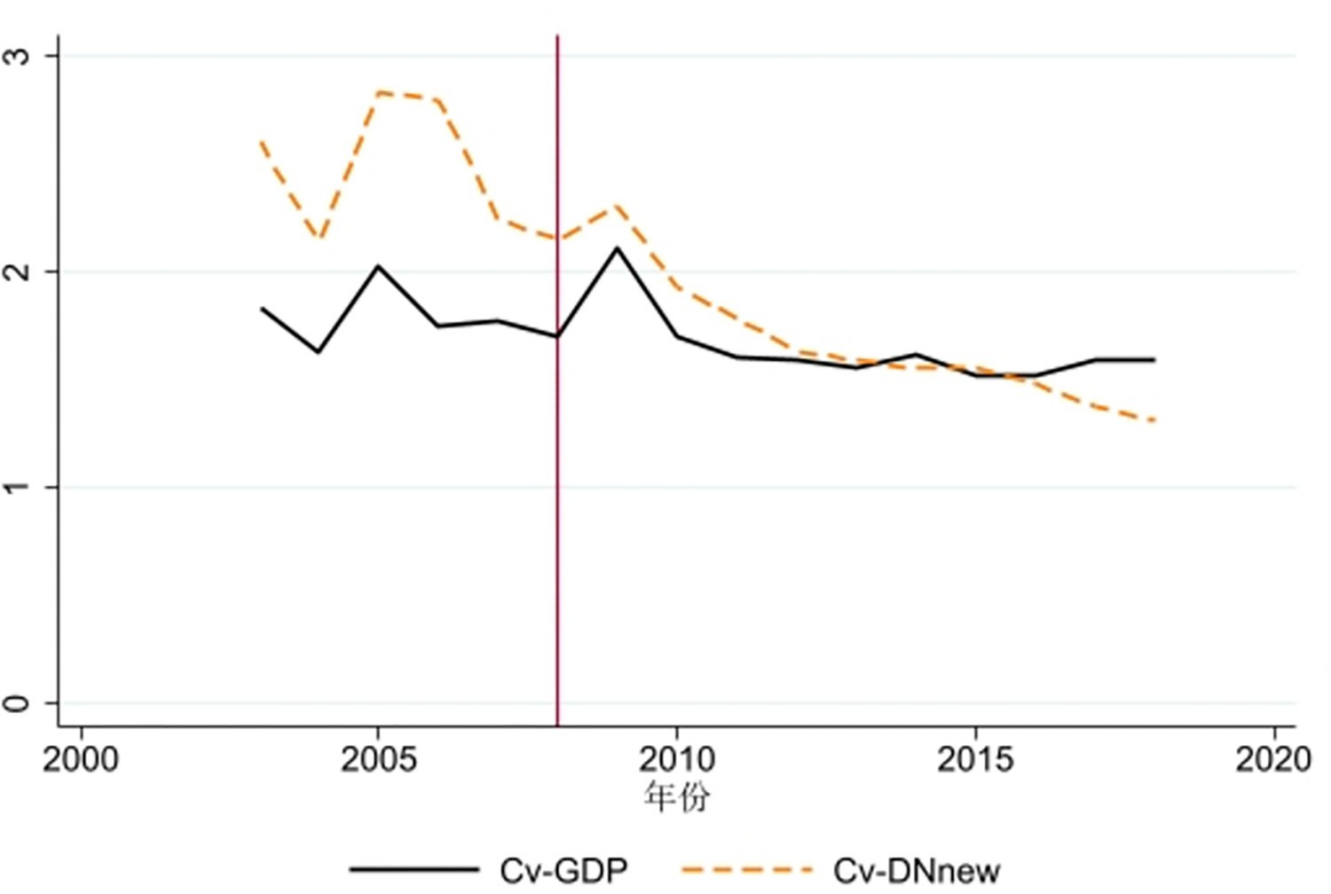
Economic disparity trends in the Yangtze River Delta region before and after the inauguration of high-speed rail.

This study uses both urban nighttime light intensity and GDP as measures of urban economic activity, and employs the coefficient of variation to gauge the economic disparities within the Yangtze River Delta region, resulting in two distinct indicators, as shown in Fig 5. It is observed that the trend in the coefficient of variation remains largely consistent whether GDP or nighttime light is used as a measure of economic growth. Prior to the inauguration of the high-speed rail in 2008, a significant economic disparity was apparent in the Yangtze River Delta region. However, following the rail’s opening, there has been a general decline in the coefficient of variation. Interestingly, the economic disparity within the region actually increased in the year of the high-speed rail’s operation and the subsequent year. Yet, two years post-operation, the high-speed rail evidently contributes to narrowing the regional economic disparity, with its effect increasing over time. This reveals that the impact of high-speed rail on regional economic disparity has a significant lag effect, which corroborates the conclusion drawn in section 5.3.2. A plausible explanation for this finding is that in the initial two years following the launch of the high-speed rail, its role in enhancing the mobility of production factors and the dissemination of information wasn’t apparent, hence its effect on reducing regional economic disparity was not fully realized. However, as time progresses and transportation infrastructure improves, the economic disparity among regions gradually diminishes, with the central and peripheral areas experiencing converging economic growth. This is in agreement with the view proposed by Nian [58], suggesting that large-scale transportation infrastructure, especially high-speed rail, has a cumulative time effect, meaning that it requires time to fully stimulate urban economic growth. This is because the Neoclassical Theory of Regional Equilibrium Development posits that the tendency for factor mobility will lead to the equalization of average returns, with regional economic growth ultimately demonstrating convergence. As the density of the high-speed rail network increases, the connection between central and peripheral areas becomes more frequent, facilitating the exchange of knowledge, information, and technology, and sparking a “knowledge spillover”. This provides a more convenient channel for peripheral regions to learn from central regions, thereby enhancing their innovative capacity, boosting their economic growth disparity with central regions.

This conclusion addresses the academic debate regarding the impact of high-speed rail on regional economic disparities. The primary cause of this dispute lies in the duration of the research period. That, in the initial stages of high-speed rail operation, it appears to exacerbate regional economic disparities, creating a polarizing effect. However, with the gradual expansion and refinement of the high-speed rail network, it eventually leads to the synchronization and convergence regional economic development. This finding serves as a reminder to scholars not to solely focus on the short-term of high-speed rail on socio-economic aspects. Instead, it is crucial to pay attention to the medium and long-term effects of high-speed rail on economic development.

## 9. Conclusion and policy recommendations

The key findings of this study are as follows: (1) High-speed rail operations have a significant promoting effect on regional economic growth, and this effect exhibits a lagged nature. (2) Regarding the analysis of heterogeneity in urban size, the study concludes that high-speed rail operations do not have a significant impact on the economic growth of medium-sized cities. However, they have a strong stimulating effect on the economies of large and mega-cities. Moreover, the impact becomes more pronounced as the city size increases. In terms of urban industrial structure, high-speed rail operations have a significant impact on the economic growth of cities that are primarily dominated by the secondary and tertiary industries. This effect may be attributed to the sensitivity of these industries to face-to-face service requirements. (3) Based on the analysis of transmission mechanisms, high-speed rail promotes regional economic growth primarily through three channels. Firstly, high-speed rail facilitates a shift towards a green economic growth model by promoting low-carbon emissions, which significantly contributes to economic growth in the region. Secondly, high-speed rail enhances labor force agglomeration effects, stimulating regional economic growth. Thirdly, high-speed rail improves the level of technological innovation, providing intrinsic growth momentum for the regional economy.(4) As the high-speed rail network gradually improves, the operation of high-speed rail ultimately leads to a trend of convergence or harmonization in regional economies.

This article proposes the following recommendations: The government should prioritize the development and expansion of high-speed rail networks, with a focus on strengthening regional connectivity and promoting economic integration. In addition to high-speed rail, it is crucial to invest in complementary infrastructure such as roads, airports, and logistics facilities. This will optimize the overall transportation system and enhance the efficiency of the high-speed rail network. Moreover, the Yangtze River Delta region should adopt various preferential policies to enhance its attractiveness for urban talents and increase investment in scientific and technological innovation to enhance the soft power of economic growth. Additionally, prioritizing ecological conservation and pursuing green development are crucial in promoting sustainable economic growth in China. Actively guiding the large-scale construction and application of green industries, such as high-speed rail, will provide inexhaustible momentum for achieving the "dual-carbon" goals on schedule.

## Supporting information

S1 File Excel 1: Fig 1 data. The economic growth trends of different regions in the Yangtze River Delta from 2003 to 2020. Excel 2: Figs 2 and 3 data. The parallel trend test. Excel 3: Fig 4 data. Placebo test. Excel 4: Economic Disparity Trends in the Yangtze River Delta Region Before and After the Inauguration of High-Speed Rail.
(ZIP)

S2 File These data were used to generate Tables 3–10. (ZIP)
